# Multimodal Approach for Lower Leg Liposculpture: A Case Series and Lessons Learned

**DOI:** 10.1093/asjof/ojag134

**Published:** 2026-06-26

**Authors:** Libby R Copeland-Halperin, Lauren O’Brien, Michelle Copeland

## Abstract

Lower leg lipodystrophy can be distressing to patients. Liposuction can contour the ankle, calf, and medial knee areas but requires the understanding of anatomy, thorough preoperative evaluation, and diligent postoperative care. Previous studies focused on a single liposuction technique. In this preliminary report, the authors presented a multimodal technique for lower leg liposculpture for aesthetic lower leg contouring and discussed lessons learned. The authors performed a retrospective review of patients undergoing bilateral liposculpture contouring using a combination of laser, power-assisted, and fine-cannula liposuction of the ankles and calves with or without medial knee contouring between 2021 and 2025. Patients undergoing unilateral procedures, revision surgery, or surgery for lipedema were excluded, as were cases involving liposuction limited to the ankles or knees. Of 40 cases undergoing lower leg liposuction procedures, 30 involved liposculpture of the ankles, calves, and medial knees. The average lipoaspirate was 793 ± 418 cc (range, 300-2700 cc) from each leg. The mean operative duration was 115 ± 33 min (range, 72-200 min), including concurrent procedures. The mean follow-up was 4.2 ± 4.3 months (range, 1 week to 20.5 months). Three patients experienced complications: one developing skin hyperpigmentation, one with scar thickening, and one with pressure injury with cellulitis and prolonged edema. The application of lower leg liposuction has been limited by concerns about complications. The authors present a multimodal approach to lower leg liposculpture. With appropriate preoperative assessment, patient counseling, and postoperative care, this approach can improve lower leg aesthetics with limited adverse events.

Level of Evidence: 4 (Therapeutic)

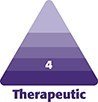

Patients with lower leg lipodystrophy may report dissatisfaction with the appearance of their lower legs, affecting self-esteem or limiting clothing options, commonly avoiding short pants, skirts, or boots. The cosmetic problem is typically resistant to dietary and exercise interventions.^1-4^

Lower leg liposuction can contour the ankle, calf, and medial knee areas but requires precise understanding of the anatomy. Furthermore, lower leg liposuction represents a unique surgical challenge because of the risks of contour irregularities, damage to surrounding nerves and lymphatics, prolonged edema, and deep vein thrombosis. In the 1920s, Dujarrier reported a patient in whom curettage to contour the lower legs of a ballerina caused transection of a major artery, resulting in amputation.^5^ Following the advent of suction-assisted lipectomy by Illouz in the 1970s, liposuction of the lower leg was increasingly performed.^2,3,6,7^

The application of the technique has been limited because of concerns about potential complications, such as persistent hyperpigmentation, contour irregularities, and prolonged edema leading to poor patient satisfaction.^2,3,6,8^ Previous reports noted that the lower leg consists of a layer of dense fibrous tissue with lymphatics that can result in palpable and/or visible irregularity, warranting caution when performing lower leg liposuction.^1,7,9,10^ Techniques have evolved, but limited details about specific methodology have been reported.

Most studies have focused on a single liposuction technique, such as standard or power-assisted liposuction. We describe our multimodal approach using a combination of external ultrasound, laser liposuction, power-assisted liposuction, and conventional liposuction and discuss lessons learned from treatment in this nuanced area. We have found this combined technique to be effective at contouring and creating a shapely lower leg while helping to reduce tissue trauma and postoperative edema.

## METHODS

We performed a chart review of all patients undergoing bilateral liposculpture contouring of the ankle and calf regions for aesthetic concerns between 2021 and 2025. Biomedical Research Alliance of New York IRB approval was obtained, and principles of the Declaration of Helsinki were adhered to with patient consent for the use of data for research purposes. We included patients who underwent medial knee liposuction concurrently with calf and ankle liposuction but excluded those undergoing only ankle or medial knee liposuction, unilateral procedures, revision following surgery performed elsewhere, or surgery for lipedema. Charts were reviewed for patient demographics, operative details, follow-up, and complications. Complications were defined as postoperative skin hyperpigmentation of the calf or ankle distinct from the surgical incisions, venous thromboembolism (VTE) or diagnostic testing to exclude VTE, infection, or patient dissatisfaction. Descriptive statistics are reported as the mean ± standard deviation and range.

### Preoperative Evaluation, Surgical Technique, and Postoperative Care

Preoperative medical evaluation and indicated testing included a medical history of tobacco use, oral contraceptive drugs or hormone replacement, a Caprini score assessment, previous lower leg surgery (including for varicose veins or orthopedic procedures with or without prosthetic hardware), or trauma. A history of lower leg surgery or trauma was not an absolute contraindication to liposculpture and was evaluated on a case-by-case basis. Similarly, the presence of varicose veins was not a contraindication to surgery but may have influenced preoperative counseling regarding the potential for increased postoperative ecchymosis and edema. Hormone replacement therapy and oral contraceptive medications were stopped at least 2 weeks preoperatively, and smoking cessation was required for at least 4 weeks perioperatively. Evaluation also addressed lower leg edema, varicosities, lymphedema, or lipedema. Assessment included examination of subcutaneous tissue thickness using a pinch test and gastrocnemius muscle activity and caliber, as well as photography. Patients were examined in the standing position to better assess skin irregularities, muscle tone, and contour, including the medial knee area. Legs were marked with the patient standing with attention to subcutaneous tissue thickening in the calf transition zone, ankle, and medial knee, as well as the anterior lower leg where a thin layer of fat is common (Video 1).

Procedures were performed as outpatient surgery, and all patients underwent the following multimodal technique. In the operating room, patients were positioned prone and with tumescent liposuction under intravenous sedation administered by board-certified anesthesiologists. An incision was placed on either side of the ankle and upper calf. A tumescent solution was instilled in the first leg, and SmartLipo laser liposuction (Cynosure Inc., Westford, MA) performed in the subcutaneous plane for 9 min at 10,000 J, 40 Hz, and 18 W, and Silberg EUA external ultrasound (Wells Johnson Company, Tucson, AZ) was performed for 3 min using a 10 cm^2^ applicator at 1 MHz and 30 W with sterile gel lubricant. Power-assisted liposuction was followed by standard liposuction using 3 and/or 4 mm cannulas from proximal to distal and distal to proximal along the entirety of the anterior and posterior calf and ankle regions for circumferential contouring (Video 2). Attention was directed to the transition zone from the calf to the ankle and the posterior aspect of the ankle around the Achilles tendon, avoiding overreduction over the anterior tibial surface, where there is typically a thin layer of subcutaneous fat.^4,11^ A thin layer of subcutaneous tissue should remain over the gastrocnemius muscle to reduce the risk of contour irregularities and to avoid an overly sculpted appearance. The majority of lipoaspirate was removed using power-assisted liposuction, whereas additional contouring and refinement were performed with standard liposuction. However, the amount of liposuction performed with each modality varied with the degree of lipodystrophy and aesthetic goals. For medial knee liposuction, this area was accessed through the medial posterior calf incision. Once the desired aesthetic contour was achieved, incisions were closed with 4-0 Vicryl suture (Johnson & Johnson Medical GmbH, Norderstedt, Germany), and the leg elevated while a similar procedure was performed on the opposite leg. Compression stockings were applied at the conclusion of the procedure.

Patients were encouraged to ambulate immediately after surgery and recovery from anesthesia. Compression garments were kept in place for 1 week, then removed for showering for 1 to 2 weeks, and then worn only during the daytime for several weeks, depending on postoperative ecchymosis, edema, and type of work patients performed. Activity was increased as tolerated 2 to 3 weeks postoperatively, depending on the degree of postoperative ecchymosis and edema, and patients were instructed on specific postoperative exercises, topical creams, and scar massage. Follow-up assessments were scheduled at 1, 3, 6, and 12 weeks, 6 months, and 1 year postoperatively, with lymphatic massage occasionally performed during the first postoperative visit and additionally at weekly intervals at surgeon and patient discretion.

## RESULTS

**Figure 1.**
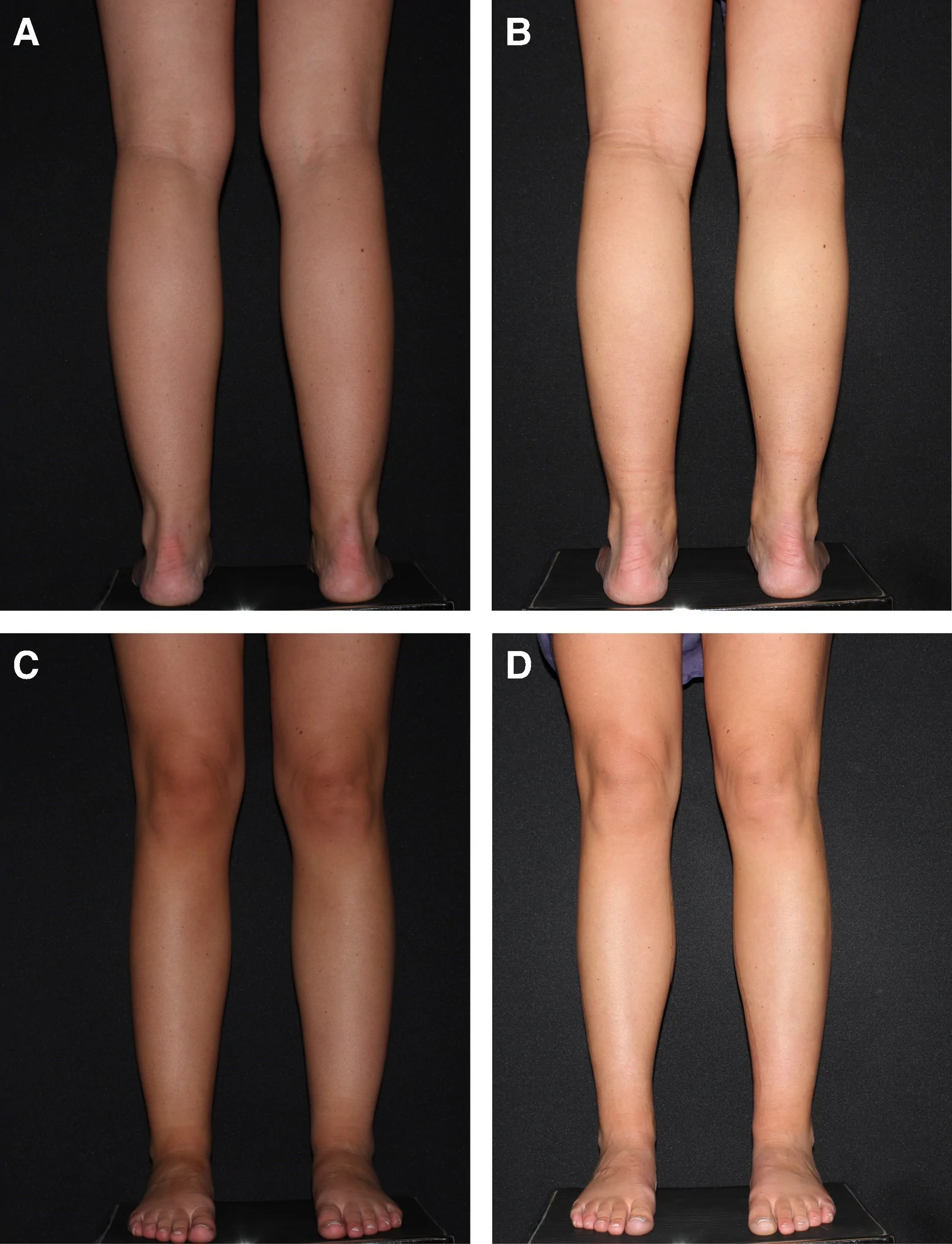
Photographs of a 30-year-old female patient (A, C) before and (B, D) 14 months after liposuction of the bilateral calf, ankle, and medial knee with 600 cc lipoaspirate from the left and right legs each.

**Figure 2.**
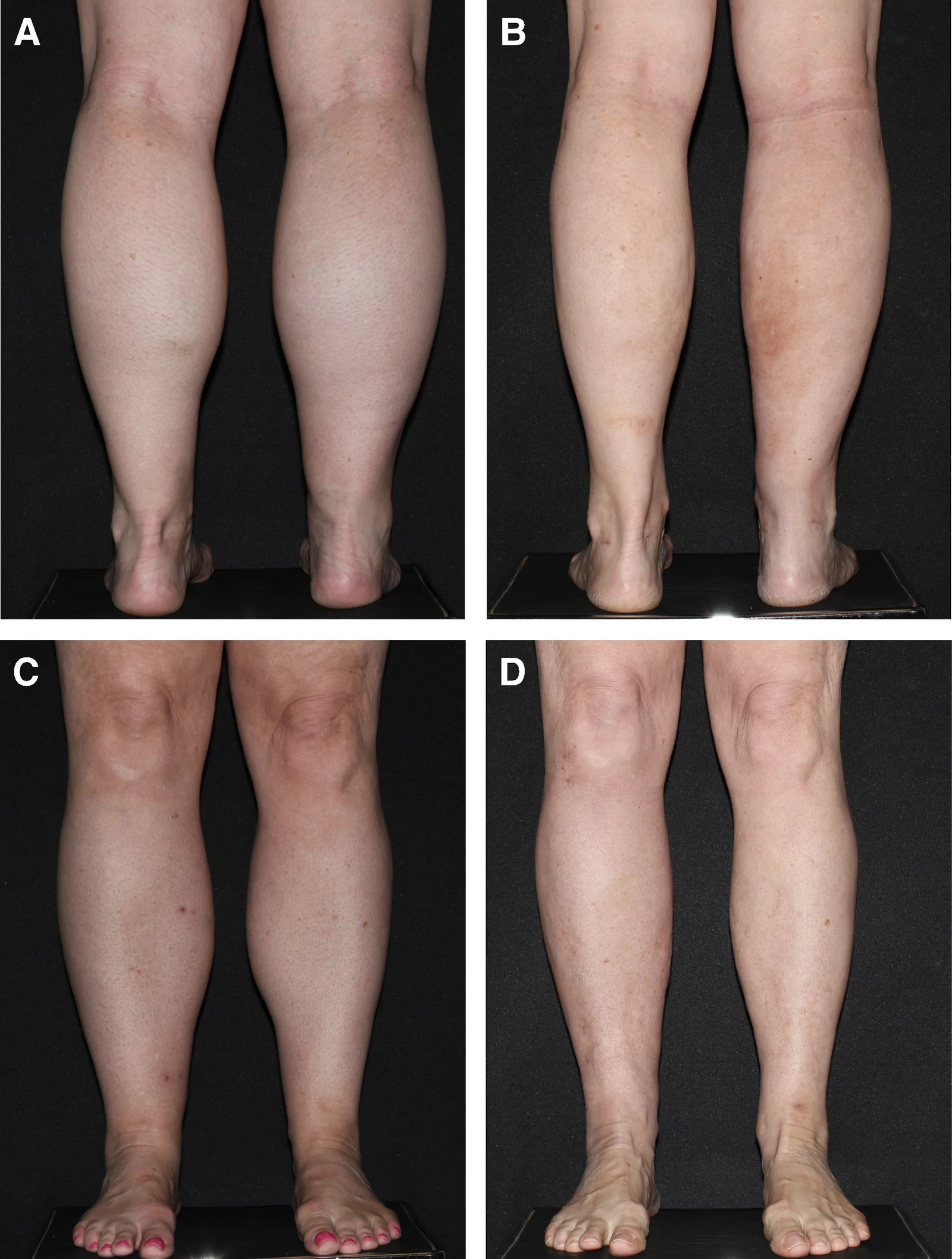
Photographs of a 64-year-old female patient (A, C) before and (B, D) 19 months after liposuction of the bilateral calf, ankle, and medial knee with 700 and 900 cc of lipoaspirate from the left and right legs, respectively. This patient developed skin hyperpigmentation for which she was subsequently treated with topical and laser treatments.

Forty patients (ages 21-71 years, mean 43 years) were included. All were women dissatisfied with the appearance of their calves and ankles and, in 30 cases (75%), with the medial aspect of the knees as well. The average BMI was 24.1 kg/m^2^ (range, 18-37.8 kg/m^2^). Patient demographics, comorbidities, and operative details are described in Tables 1 and 2. For each patient, laser liposuction was performed for 9 min at 40 Hz/18 W/10,000 J. On average, 512 ± 182 cc (range, 250-1000 cc) and 513 ± 178 cc (range, 250-1000 cc) of tumescent were instilled into the left and right legs, respectively. The average lipoaspirate was 794 ± 435 cc (range, 325-2700 cc) and 774 ± 406 cc (range, 300-2500 cc) from each leg. The mean operative time was 115 ± 33 min (range, 72-200 min), including for patients undergoing other procedures concurrently. The mean follow-up was 4.2 ± 4.3 months (range, 1 week to 20.5 months). Preoperative and postoperative results are shown in Figures 1 and 2.

**Table 1.** Patient Demographics

		Comments
Patient age, mean (range)	43 years (21-71 years)	—
Race, *n* (%)	35 Caucasian patients (90%) 4 Black patients (10%)	—
Personal or family history of VTE, *n* (%)	2 patients (5%)	1 patient with a personal history of unprovoked DVT 1 patient with a family history of DVT
Previous lower leg procedures or trauma, *n* (%)	5 patients (12.5%)	1 patient with a history of bilateral knee replacement 2 patients with a history of unilateral anterolateral cruciate ligament repair 2 patients with a history of bilateral lower leg varicose vein ablation 1 patient with a history of bilateral lower leg sclerotherapy
Current or former tobacco use, *n* (%)	2 patients (5%)^a^	—
Current OCP or HRT, *n* (%)	9 patients (22.5%)^b^	—
BMI, mean (range)	24.1 kg/m^2^ (18-37.8 kg/m^2^)	—

DVT, deep vein thrombosis; HRT, hormone replacement therapy; OCP, oral contraceptive pills; VTE, venous thromboembolism.

^a^Tobacco cessation 4 weeks perioperatively.

^b^OCP or HRT stopped 2 to 4 weeks perioperatively.

**Table 2.** Intraoperative and Postoperative Details

		Comments
Tumescent Left leg, mean ± SD (range) Right leg, mean ± SD (range)	512 ± 182 cc (range, 250-1000 cc) 513 ± 178 cc (range, 250-1000 cc)	—
Total liposuction lipoaspirate Left leg, mean ± SD (range) Right leg, mean ± SD (range)	794 ± 435 cc (range, 325-2700 cc) 774 ± 406 cc (range, 300-2500 cc)	—
Medial knee liposuction, *n* (%)	30 patients (75%)	—
Other concurrent procedure,^a^ *n* (%)	8 patients (20%)	1 circumferential thigh liposuction 2 inner thigh liposuction 1 Thermage^©^ and radiofrequency microneedling to thighs and knees 1 liposuction of anterior knees 1 fat transfer to breasts 1 liposuction of bilateral axilla 1 liposuction of the trunk
Operative time, mean ± SD (range)	115 ± 33 min (72-200 min)	—
Follow-up, mean ± SD (range)	4.2 ± 4.3 months (1 week to 20.5 months)	—
Complications, *n* (%)	3 patients (7.5%)	1 patient with postoperative skin hyperpigmentation secondary to ecchymosis 1 patient with hypertrophic scarring 1 patient with postoperative pressure wound with cellulitis and asymmetric leg swelling (negative for DVT)

DVT, deep vein thrombosis; SD, standard deviation.

^a^Concurrent procedures involving other areas of the body, other than/in addition to medial knee liposuction.

Eight patients (20%) underwent concurrent procedures involving other areas at the time of lower leg contouring, including circumferential thigh liposuction (1 patient), liposuction of the inner thighs only (2 patients), liposuction of the anterior knees (1 patient), autologous fat transfer to the breasts (1 patient), liposuction of the trunk (1 patient), bilateral axillary liposuction (1 patient), and Thermage^©^ (Solta Medical Inc., Bothell, WA) and radiofrequency microneedling (Lutronic Inc., Billerica, MA) on the thighs (1 patient). Three patients (7.5%) underwent botulinum toxin injections (mean 54 units injected per leg) to treat hypertrophic gastrocnemius muscles in conjunction with lower leg liposculpture. Five (12.5%) had undergone previous procedures on the knee, calf, or ankle region, including varicose vein procedures (3) and knee surgery (3).

Three patients (7.5%) experienced complications. One developed skin hyperpigmentation likely related to postoperative ecchymosis that improved with topical and laser treatment, another developed scar thickening requiring triamcinolone injection, and a third developed a pressure injury with cellulitis on postoperative Day 23 that required debridement and oral antibiotic medication, as well as postoperative edema; duplex ultrasound was negative for deep vein thrombosis, which was not detected in any patient postoperatively.

## DISCUSSION

The senior author (M.C.) began performing lower leg liposculpture in 2001 using longer durations of laser liposuction, as well as ultrasonic liposuction, in addition to power-assisted and fine-cannula liposuction. Through refinement in technique, we now use shorter durations of laser liposuction, and ultrasonic liposuction is no longer employed because of potential skin and soft tissue injury and limited benefit when assessed subjectively by the senior author. Thigh liposuction performed concurrently with lower leg liposuction was sometimes associated with increased postoperative edema and discomfort, so our current practice is to stage liposuction of the thighs and lower legs.

Our current technique involves a combination of laser liposuction, power-assisted liposuction, and conventional liposuction, as well as external ultrasound. External ultrasound can loosen intercellular connections and facilitate removal of adipose tissue with less cannula resistance, tissue trauma, and postoperative edema and faster postoperative recovery.^12,13^ When performed in conjunction with external ultrasound, we found that laser liposuction facilitates liposuction of the fibrous subcutaneous tissue in the lower leg, reduces resistance to the suction cannula, and makes the procedure technically easier to perform. Laser liposuction may also reduce tissue trauma and postoperative edema.^14^ When performed for aesthetic indications, liposculpture of the lower leg requires appreciation of contour in the context of the entire leg.^2,3,8,9,10^ Although in some patients, lipodystrophy may be isolated to the lower legs, others have lipodystrophy of the thighs, as well. Lower leg shape is determined by several factors, including underlying bone structure, musculature, and subcutaneous tissue.^6^ Thorough preoperative evaluation is essential to identify factors contributing to an unshapely lower leg.

Examining patients while they stand with feet flat on the floor and on their toes facilitates the evaluation of the musculature, skin irregularities, and contour.^11^ Patients with subcutaneous tissue thickening or maldistribution of fat and realistic expectations are typically appropriate candidates for liposculpture contouring. The goal is not “skinny legs,” but rather a pleasing curvature at the calf that tapers to a contoured ankle. Transition zones depend upon individual anatomy.^15,16^ For patients with hypertrophic gastrocnemius muscles, botulinum toxin can reduce muscle tone, with care taken to avoid muscle weakness; in some instances, patients are treated with a combination of botulinum toxin and liposculpture.

Liposuction should be performed with a sense of overall lower leg aesthetics in mind to create a gradual tapering and contour, and the lower leg should not be uniformly reduced in all areas.^17^ It is important to preserve a thin layer of subcutaneous adipose tissue so the skin is not adherent to muscle and the lower leg does not appear overly muscular, particularly in the calf.^1,9^ Leaving a layer of subcutaneous fat over the musculature also helps to decrease the risk of contour irregularities. More aggressive liposuction can be performed at the ankle, as an aesthetically pleasing lower leg has minimal subcutaneous adipose tissue on either side of the ankle and overlying the Achilles tendon and around the calcaneus. However, caution is required when performing liposuction in this region, because of the thin skin and close proximity of the saphenous vein. In contrast to the other areas of the body, skin laxity is not typically a concern in the lower leg.^1,3,4,15^ We have refined our technique to reduce the duration of laser liposuction and eliminate the use of ultrasonic liposuction to reduce the risk of tissue injury and contour irregularity.

Use of tumescent liposuction and discretely placed incisions can also help optimize outcomes. Although previous reports typically described a large number of incisions, we have found that 2 small incisions on either side of the ankle and on either side of the proximal calf provide sufficient access to contour the entire lower leg, ankle, and medial knee areas, while limiting scarring.^1,2,4,6,11,18,19^ The procedure is performed using a tumescent technique to facilitate hemostasis, reduce pain, minimize risk of lymphatic injury, and reduce postoperative ecchymosis compared with dry techniques.^20^ Although previous reports raised concerns regarding anatomic distortion with tumescent infiltration, we have not encountered this.^18,21^

Three patients (7.5%) in our cohort experienced complications that included skin hyperpigmentation likely related to hemosiderin deposits secondary to postoperative ecchymosis that improved with a combination of topical and laser treatments (Figure 2), scar thickening that was treated with triamcinolone injections, and soft tissue pressure injury with cellulitis requiring debridement and oral antibiotic medication. Duplex Doppler ultrasound was negative for deep vein thrombosis, and asymmetrical limb edema resolved. Impaired postoperative sensation and prolonged pressure likely contributed to pressure-induced soft tissue injury. We did not encounter significant contour irregularity, and thorough preoperative assessment and refinement in technique have made this no more frequent than with liposuction of other body regions.

Postoperative follow-up ranged from 1 week to 20.5 months. We encourage follow-up at 1, 3, 6, and 12 weeks, 6 months, and 1 year postoperatively. Although patients are always advised to return regularly postoperatively and the expected duration of follow-up is discussed preoperatively, some were unable to return for in-person visits or were lost to follow-up despite efforts to contact them. Because of the potential for complications in lower leg contouring because of prolonged postoperative edema, close postoperative follow-up may be more important with this procedure than with liposuction of other body areas.

VTE is of particular concern with lower leg liposuction because of postoperative edema and reduced mobility. No patients in our series experienced clinical evidence of VTE, although one underwent ultrasound evaluation to exclude this postoperatively. During the initial consultation, patients were screened for risk factors for VTE, as those at high risk are not appropriate candidates for the procedure. Patients were instructed to cease smoking and interrupt oral contraception or hormone replacement therapy as mentioned above. Although patients are not immobilized postoperatively, and there are limited data to support these measures, these recommendations extend from other lower extremity surgical procedures.^22^ Elevated BMI is a risk factor for VTE, and although some authors restrict the procedure to patients with BMI <30, we do not enforce a strict cutoff. Rather, BMI is considered a potential risk factor in the context of other comorbidities and body habitus. Patients are placed in compression garments at the end of the procedure, and postoperative ambulation is encouraged beginning on the day of surgery. Additionally, although previous reports describe performing this procedure under general anesthesia, we performed all procedures under intravenous sedation, which is associated with a lower risk of VTE compared with general anesthesia.^15,18,23-27^ Although sequential compression devices cannot be applied intraoperatively, the legs are mobilized throughout the procedure, and compression garments are applied immediately postoperatively. We have not found routine postoperative chemical chemoprophylaxis necessary unless the patient has risk factors for VTE.

Postoperative edema can persist for 6 to 12 months, due mainly to dependency of the lower legs and potential lymphatic disruption, although we have not found prolonged postoperative edema to be a significant problem with our technique.^1,7,18^ There are several potential explanations for this. We avoid performing extensive liposuction on the upper and lower legs concurrently to reduce the risk of prolonged postoperative edema and VTE. The use of tumescent liposuction reduces the risk of lymphatic injury, and laser liposuction and external ultrasound may also reduce the risk of soft tissue trauma and prolonged postoperative edema.^12-14,20^ Leg elevation and compression postoperatively are also essential. Leg elevation is performed intraoperatively after completing the first leg, and appropriate postoperative compression is initiated immediately postoperatively and continued continuously for the first week postoperatively and for several weeks thereafter based on the degree of ecchymosis and edema present. We have also found lymphatic massage to play an important role in limiting postoperative edema and speeding recovery. However, we have not performed a randomized trial directly comparing the incidence and severity of postoperative edema with and without these elements.

Previous studies described postoperative pain as a limitation of this procedure.^1,7,9,18^ Although not formally assessed, we have not found it more painful for patients than liposuction of other body areas. Discomfort is due mainly to swelling, and although analgesic medication is prescribed postoperatively, patients are counseled to apply ice and elevate their legs frequently during the initial postoperative period. Intraoperatively, the legs are elevated as soon as the procedure is completed on each side, and thigh-length compression garments are applied at the end of the procedure and remain in place for the first week to control swelling. Lymphatic massage is also incorporated into the early postoperative period as described.

Although not formally assessed in this study, anecdotally, patient satisfaction was generally high, consistent with the literature.^7,9^ This may be because of self-selection, because patients were typically bothered by their lower leg appearance for many years and were unable to improve leg contour through nonsurgical methods. Thorough preoperative counseling is essential regarding the risks of contour irregularity, scarring, hyperpigmentation, and prolonged edema, and those who do not accept these risks are not suitable candidates for this procedure.

To minimize heterogeneity in technique and potential confounding, we included only patients undergoing procedures between 2021 and 2025 for this analysis. Patients undergoing lower leg liposuction for lipedema or revision or secondary lower leg contouring were also excluded to limit heterogeneity and confounding. Although a complete review of lipedema is beyond the scope of this article, patients with lipedema were excluded because of distinct characteristics, risks, and considerations.

This study is limited by small sample size and length of follow-up in part because of a focus on patients undergoing primary lower leg liposculpture for aesthetic indications and limiting the time window to a 5-year period. As noted, although patients were advised to follow-up at regular intervals postoperatively, some were unable to do so. The lack of a formal patient satisfaction survey is also a limitation of this study, but for this type of retrospective report, a survey would have been prone to response bias and other confounding. Another limitation is the retrospective design. Additionally, data presented here represent our experience and analysis is limited by lack of objective outcomes data or formal aesthetic assessment. As with any surgical procedure, results are subject to instrument-related variability and surgeon experience that may limit generalizability. Future research is planned to more formally evaluate the aesthetic outcomes of our technique. Ultimately, larger, multisurgeon studies are warranted to further evaluate the optimal technique for lower leg liposculpture.

## CONCLUSIONS

Widespread application of lower leg liposuction for contouring has been limited by concerns about the risk of complications such as prolonged postoperative edema, deep vein thrombosis, skin hyperpigmentation, or contour irregularities. We describe experience using a multimodal approach and discuss methods to minimize complications and enhance patient satisfaction. Combined with appropriate preoperative assessment, thorough patient counseling, and diligent postoperative care, this preliminary report found that this technique can be a safe and effective way to create a shapely, aesthetic lower leg.
