# Correction: Multimodal Approach for Lower Leg Liposculpture: A Case Series and Lessons Learned

**DOI:** 10.1093/asjof/ojag183

**Published:** 2026-09-22

This is a correction to: Libby R Copeland-Halperin, Lauren O’Brien, Michelle Copeland, Multimodal Approach for Lower Leg Liposculpture: A Case Series and Lessons Learned, *Aesthetic Surgery Journal Open Forum*, Volume 8, 2026, ojag134, https://doi.org/10.1093/asjof/ojag134

The following change has been made to the originally published paper. A errored credential was corrected in the disclosure section.

